# Microphthalmia/Transcription Factor E (MiT/TFE) Pathways in Pulmonary Diseases: Current Evidence and Emerging Mechanisms

**DOI:** 10.3390/cells15080719

**Published:** 2026-04-18

**Authors:** Priyanka Singh, Evans Kwabena Abor, Wei Shi

**Affiliations:** Division of Pulmonary, Critical Care and Sleep Medicine, Department of Internal Medicine, University of Cincinnati, Cincinnati, OH 45267, USA; aborek@mail.uc.edu

**Keywords:** MITF, TFEB, TFE3, cancer, lung injury, asthma, lysosome–autophagy pathway, BHD

## Abstract

**Highlights:**

**What are the main findings?**

- The MiT/TFE family transcription factors, are increasingly recognized for their context-dependent role in conditions such as asthma, lung cancer, Birt–Hogg–Dube syndrome (BHD), lysosomal storage diseases, chronic obstructive pulmonary diseases (COPD)/emphysema, and acute lung injury with fibrotic remodeling.
- The MiT/TFE signaling pathway provides protective effects in lung injury and inflammation; on the other hand, it also contributes to tumor progression and resistance to therapies.

**What are the implications of the main findings?**

- This review highlights the evidence associated with TFEB, TFE3 and MITF in pulmonary diseases and underscores the potential for future mechanistic understanding and the development of MiT/TFE-targeted therapies in pulmonary diseases.

**Abstract:**

The MiT/TFE family transcription factors play a critical role in lysosomal biogenesis, autophagy, mitochondrial turnover and lipid catabolism by regulating the Coordinated Lysosomal Expression and Regulation (CLEAR)gene network. The dysregulation of MiT/TFE activity has been implicated in the onset and progression of cancer and neurodegeneration, but its functions in association with pulmonary diseases remain poorly understood. In this review, we systematically summarize the findings from human pulmonary diseases and associated genetic disorders, such as asthma, cancer, Birt–Hogg–Dube (BHD) syndrome, and lung injury models that implicate MiT/TFE dysregulation in pathogenic progression. We also discussed MiT/TFE regulation and signaling through pathways involving mTORC1, AMPK, and lysosomal stress in different cellular contexts. Finally, we discussed significant mechanistic gaps, such as the absence of in vivo models targeting the combined activity of TFEB and TFE3 in disease progression and prevention. In conclusion, these insights seek to offer a comprehensive framework for understanding MiT/TFE signaling in human lung diseases and could present a promising opportunity for directing future mechanistic and translational research.

## 1. Introduction

The microphthalmia/transcription factor E (MiT/TFE) family comprises a small group of evolutionarily conserved transcription factors that play central roles in cellular adaptation to metabolic and environmental stress [1,2]. Members of this family belong to the basic helix–loop–helix leucine zipper (bHLH-LZ) superfamily of transcription factors, which includes regulators such as MYC, MAX, SREBP, USF, MLX, and AP4 [3,4,5,6]. In vertebrates, the MiT/TFE family consists of four closely related proteins: microphthalmia-associated transcription factor (MITF), transcription factor EB (TFEB), TFE3, and TFEC. These proteins form homo- or heterodimers via their leucine zipper domains and bind DNA through a conserved basic region that recognizes E-box motifs (CANNTG) and/or M-box motif (CATGTG) and modified E-box motif containing CLEAR element, a palindromic 10-base-pair motif (GTCACGTGAC) within their target gene promoters [7,8,9].

TFEB was first identified as a transcriptional regulator of the CLEAR network, a discovery that established MiT/TFE proteins as master regulators of lysosomal biogenesis and autophagy [8]. Subsequent studies demonstrated that TFE3 and MITF can also bind CLEAR elements, highlighting functional redundancy and cooperation among family members [1,10,11,12]. These transcription factors coordinate a broad transcriptional program that extends beyond lysosomal gene expression to genes involved in autophagy initiation, autophagosome formation and trafficking, lysosome–autophagosome fusion, extracellular vesicle trafficking and selective degradation pathways such as mitophagy [10,11,13,14,15,16]. Through these functions, MiT/TFE proteins promote cellular catabolic and recycling processes, thereby supporting metabolic homeostasis under nutrient deprivation, organelle damage, and other stress conditions. In addition to this, MiT/TFE factors have also been implicated in nutrient sensing, energy metabolism, mitochondrial biogenesis, oxidative and endoplasmic reticulum stress responses, innate immunity and inflammation, cell fate determination, aging, and tissue-specific differentiation programs [17,18,19,20,21,22,23,24,25,26,27,28,29,30,31,32,33].

At the molecular level, MiT/TFE protein activity is tightly regulated by post-translational modifications, most prominently phosphorylation, which integrates upstream signals from nutrient-, stress- and growth factor-sensing pathways. Under nutrient-replete conditions, the mechanistic target of rapamycin complex 1 (mTORC1) acts as a central negative regulator of TFEB and TFE3 by phosphorylating conserved serine residues that promote 14-3-3 mediated cytoplasmic retention [34,35,36]. Conversely, nutrient starvation, lysosomal dysregulation, mitochondrial dysfunction, and calcium signaling induce MiT/TFE protein dephosphorylation, nuclear translocation, and transcriptional activation of the downstream transcriptional program. Other post-translational modifications include ubiquitination, acetylation, SUMOylation, and dephosphorylation, which are also implicated in the fine-tuning of MiT/TFE protein stability and activity as mTORC1-independent mechanisms in a tissue- and context-dependent manner.

Dysregulation of MiT/TFE signaling is associated with a wide spectrum of human diseases. Aberrant activation of TFEB and TFE3 contributes to tumorigenesis, neurodegenerative disorders and lysosomal storage diseases. Owing to their central role in coordinating lysosomal and autophagic pathways, MiT/TFE transcription factors have emerged as attractive therapeutic targets. Here, we review the emerging role of MiT/TFE proteins in pulmonary disease and summarize recent progress in defining the mechanisms that regulate their activity in the lung.

## 2. Regulation of MiT/TFE Transcription Factors

Given that MiT/TFE family proteins are regulated through multiple layers of control, including isoform diversity, transcriptional inputs, and context-dependent signaling, it is useful to study them in detail to provide a clear framework. As summarized in Table 1, this includes (i) major isoforms and transcript diversity, (ii) key regulators, and (iii) the biological relevance of these factors across tissues and disease settings. This organization helps distinguish how structural variation and upstream regulatory networks shape the functional roles of MITF, TFEB, TFE3, and TFEC in development, cellular homeostasis, stress adaptation, and disease-associated processes.

### 2.1. MITF

**Figure 1:**
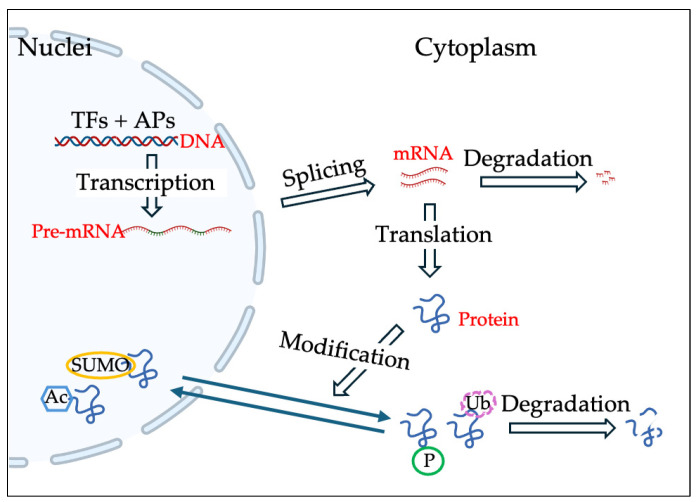
Transcriptional and post-translational regulation of MiT/TFE family. Schematic representation illustrates the key regulatory mechanisms for MIT/TFE class of protein expression and activity in general. Specific transcription factors (TFs) induce transcription from alternative promoters (APs) in different cells. The level and type of mature mRNA are then controlled by alternative splicing and miRNA-mediated degradation. The activity and turnover of produced proteins are further regulated by post-translational modifications such as phosphorylation, SUMOylation, acetylation, and ubiquitination.

The microphthalmia-associated transcription factor (MITF) is a highly conserved transcription factor that plays a critical role in melanocyte development, differentiation, and survival, as well as in melanoma pathology. The *MITF* gene is located on chromosome 3 (approximately 230,000 bp) in humans and chromosome 6 (approximately 215,000 bp) in mice, respectively [37]. Multiple protein isoforms of MITF with different N-terminal regions are generated by using alternative promoters and splicing, allowing tissue-specific function and regulation, such as different organ development, pigment production, cell metabolism, differentiation and survival, tumor growth and invasion, etc. [38,39,40]. For example, MITF-A and MITF-M are key isoforms associated with distinct biological functions, ranging from melanocyte differentiation to the survival and invasion of melanoma cells [41,42,43]. Furthermore, MITF-H is the predominant isoform expressed in cardiomyocytes and mediates cardiac hypertrophy, whereas the mast cell–specific isoform MITF-MC primarily regulates mast cell–specific target genes [44,45]. MITF expression and activity are regulated at transcriptional, post-transcriptional, and post-translational levels (Figure 1). At the transcriptional level, *MITF* is primarily regulated by transcription factors such as SOX10, PAX3, ATF4, GLI2 and BRN2 (POU3F2), which either activate or repress *MITF* expression depending on the tissue- or cellular- context. For example, PAX3 and SOX10 enhance *MITF* transcription, linking it to melanocyte viability [46,47,48], while BRN2 and GLI2 repress *MITF* in specific melanoma subpopulations [49,50]. The *MITF* promoter also contains multiple cis-acting elements and cAMP-responsive elements (CREs), where various signaling pathways, including Wnt/β-catenin, BRAF/MAPK/ERK, PI3K/AKT/mTOR, Notch, cAMP/PKA Pathway and TGF-β signaling, converge to modulate its expression and activity [36,51,52,53,54]. Post gene transcription, the 3′-untranslational region of *MITF* mRNA can bind to an RNA-binding protein CRD-BP, which protects it from miRNA (e.g., miR340)-mediated degradation, thereby increasing *MITF* mRNA stability and protein expression [55]. Post-translational modifications, such as phosphorylation [38], SUMOylation [56,57] and acetylation [58], affect MITF-driven transcriptional programs, MITF stability, and target gene selectivity [40].

### 2.2. TFEB

TFEB is the most extensively studied and best-characterized member of the MiT/TFE family due to its central role in coordinating autophagy and lysosomal biogenesis and ubiquitous expression pattern. The human *TFEB* locus spans approximately 51,000 bp on chromosome 6, whereas mouse *Tfeb* is located on chromosome 17, and extends over 55,000 bp [59]. *TFEB* gene contains seven alternative 5′-non-coding exons that generate multiple transcripts (*TFEB-A*, *TFEB-B*, *TFEB-C*, *TFEB-D*, *TFEB-E*, *TFEB-F*, and *TFEB-G*) [37,60]. These different *TFEB* transcripts exhibit a distinct tissue distribution profile, but all encode TFEB proteins with identical N-termini. Several transcription factors, such as XBP1, PGC-1α, PPARα, and MYC, regulate *TFEB* expression and its downstream pathways. XBP1, PGC-1α and PPARα activate *TFEB* expression by directly binding to the *TFEB* promoter [61,62,63], whereas MYC acts as a repressor by directly binding to MYC response elements on the TFEB promoter [64,65]. TFEB activity is also regulated through a self-regulatory positive feedback mechanism under nutrient starvation, where TFEB binds to CLEAR elements of PGC-1α and PPARα, in response, inducing TFEB expression [66]. In addition to the full-length TFEB protein, a shorter TFEB protein isoform lacking the helix–loop–helix and leucine zipper motif, generated by alternative splicing between exon 7 and exon 9, has also been reported [67]. This small ~30 KD TFEB may act as a negative regulator of the full-length TFEB.

TFEB activity is strictly regulated by its nucleocytoplasmic localization, controlled by nutrient-sensing signaling at the lysosomal surface and post-translational modifications. TFEB has more than 20 phosphorylation sites, including multiple residues phosphorylated by mTORC1, which act as a major regulator of TFEB. However, this control is not uniform across systems and appears complex and context-dependent. Under nutrient-replete conditions, mTORC1 phosphorylates TFEB at S211 and at S142 [68,69]. Phosphorylation at Ser211 promotes TFEB binding to 14-3-3 proteins, which retain TFEB in the cytoplasm [69,70]. Under nutrient-limiting conditions or impaired lysosomal function, TFEB gets dephosphorylated, disengages from 14-3-3, and translocates to the nucleus. Nuclear TFEB activates CLEAR target genes to restore lysosomal homeostasis and cellular clearance capacity [69,70,71,72]. Studies also suggest that loss of phosphorylation only at S211 is not sufficient to drive TFEB import into the nucleus, as the S211A TFEB mutant remains responsive to mTORC1 [73]. Celis et al. identified S122 as a direct mTOR phosphorylation site, and a phosphomimetic S122 substitution largely attenuates the response of TFEB to MTORC1 inhibition [73]. Together, these findings support a multistep model in which MTORC1 regulates TFEB through coordinated phosphorylation at multiple sites, rather than through S211 alone.

Growth factors, mitogens, nutrients (such as amino acids), and stress signals integrate at the lysosomal surface, and this integration tightly regulates the mTORC1-TFEB rheostat. Receptor tyrosine kinase (RTK) signaling (such as insulin or IGF) activates the PI3K–AKT pathway [74,75,76,77], and mitogens activate the RAS–RAF–MEK–ERK pathway [78,79]. Both signaling pathways converge at the TSC1–TSC2–TBC1D7 complex upstream to mTORC1, ultimately releasing TSC-mediated inhibition of RHEB [80,81]. This reduction allows RHEB-GTP to directly activate mTORC1 [82,83]. Concurrently, amino acids regulate mTORC1 by influencing their recruitment to lysosomes through RAG-A/B or RAG-C/D GTPases and the Ragulator-v-ATPase platform [84,85,86]. This process is further modulated by GATOR1/GATOR2 and nutrient sensors like Sestrins for leucine, CASTOR for arginine, and SAMTOR for methionine/S-adenosylmethionine, along with lysosomal amino acid transport and sensing components such as SLC38A9 [87,88,89,90,91,92]. Glucose and energy sensor AMPK promote TFEB pathway activation through upstream remodeling of the RAG–mTORC1 via AMPK-mediated phosphorylation of FNIP1 and suppression of FLCN–FNIP function [93]. Starvation and lysosomal stress also release lysosomal Ca^2+^ via MCOLN1/TRPML1 and activate calcineurin that dephosphorylates TFEB [94,95]. Because TFEB is a critical substrate associated with lysosomes downstream to mTORC1, the combined effects of RHEB-driven activation and RAG-mediated lysosomal positioning determine TFEB’s phosphorylation and 14-3-3 binding. This, in turn, dictates whether TFEB remains in the cytosol or enters the nucleus to activate CLEAR genes involved in lysosomal function and autophagy.

### 2.3. TFE3

TFE3, or transcription factor E3 (Transcription Factor binding to IGHM Enhancer 3), is another MiT/TFE family transcription factor that shares significant similarities with TFEB in terms of protein structure and function. TFE3 is expressed ubiquitously, with the highest expression in the placenta, lung, and adrenal gland [60]. The *TFE3* gene is located on the short arm of the X chromosome in both mice and humans (Xp11.2) [96]. With alternative transcription start sites, TFE3 is expressed as two main isoforms, a long and a short form [96,97]. Both isoforms utilize the same RAG/mTORC1-dependent regulatory mechanism and have a similar ability to trigger the expression of lysosomal and autophagic genes when activated. However, the short isoform is missing the N-terminal 105 amino acid residues, which include a phosphorylation–ubiquitin ligase recognition site that targets it for degradation by the proteasome, known as a phosphodegron. As a result, this short isoform is consistently expressed at high levels in most cells [97].

TFE3 and TFEB share several overlapping functions. TFE3 and TFEB are partially redundant in certain functions, such as inducing lysosomal biogenesis [98] and controlling CD40 ligand expression [99]. Additionally, both factors play a role in helping cells cope with stress in the endoplasmic reticulum (ER) by promoting the expression of ATF4 and other genes involved in the unfolded protein response (UPR) [26,100,101]. However, if ER stress persists, the activation of TFEB and TFE3 may lead to cell death. This occurs either by directly triggering pro-apoptotic genes like CHOP and PUMA or indirectly by increasing ATF4 levels, which can subsequently induce CHOP and PUMA expression. Collectively, these findings indicate that the TFEB/TFE3–ATF4 signaling pathway can either support cell survival or lead to apoptosis, depending upon the stress intensity and duration. MiT/TFE family proteins have also been linked to selective autophagy programs such as ER-phagy, where TFEB/TFE3 can induce the ER-phagy receptor FAM134B during prolonged starvation [27]. However, they also have their unique functions. For example, global deletion of *Tfeb* results in early embryonic lethality in mice due to placental defects, whereas *Tfe3* knockout has no abnormal phenotypes under physiological conditions. In addition to the common target gene set, they may also have their unique target genes in different cellular contexts [102].

### 2.4. TFEC

TFEC is the only member in the family that lacks the transactivation domain. The human *TFEC* gene maps to chromosome 7q31.2, while the mouse *Tfec* gene is on chromosome 6. TFEC has 3 isoforms, TFEC-A, TFEC-B and TFEC-C. TFEC-A and TFEC-B are generated by alternative transcription start sites. Using 5′-RACE in human kidney cDNA, a third transcript (TFEC-C) was identified that starts from a distinct 5′ exon (exon 1c) located between exons 3 and 4. TFEC has restricted, tissue-specific expression, with each variant displaying a distinct distribution pattern across organs. For example, TFEC-A is enriched in the testis, thymus, trachea, colon, and prostate; TFEC-B is more broadly expressed (largely absent from heart and liver); and TFEC-C is restricted to the kidney and small intestine [60].

TFEC has been reported to heterodimerize with TFE3 and antagonize TFE3-driven transactivation and transcriptional programs [103]. TFEC is expressed strongly and selectively in macrophages [104], and TFEC-knockout mice develop normally without obvious abnormalities [105]. Rehli et al. have further shown IL-4-dependent TFEC expression through STAT6 in macrophages, and loss of TFEC reduces a small set of IL-4–responsive genes, including CSF3R (the G-CSF receptor) [105]. Another study using an OVA-induced allergic asthma mouse model showed that IL-4-induced TFEC can bind the IL-4Rα promoter and boost IL-4Rα expression, creating a positive feedback loop of IL-4–TFEC–IL-4Rα that supports M2 macrophage polarization [106,107]. Altogether, TFEC and its associated functions remain poorly characterized. Therefore, additional studies are needed to define TFEC’s roles across different human tissues and cell types.

**Table 1:** Key regulators of MiT/TFE class of proteins and their biological relevance.

Family Member	Gene Locus	Major Isoforms	Key Regulators	Functions	Biological Relevance	Reference
**MITF**	Human Chr 3 Mouse Chr 6	MITF-A, MITF-B, MITF-C, MTF-C, MITF-D, MITF-E, MITF-H, MITF-M, MITF-MC, MITF-J	SOX10, PAX3, ATF4, GLI2, BRN2, Wnt/β-catenin, BRAF/MAPK/ERK, PI3K/AKT/mTOR, Notch, cAMP/PKA, TGF-β signaling	Controls development, differentiation, metabolism, survival, pigment production, and invasion in a context-dependent manner	Important in melanocyte development and melanoma, cardiomyocyte hypertrophy and mast cell activity	[36,37,48,50,54,108]
**TFEB**	Human Chr 6; Mouse Chr 17	Full length TFEB (TFEB-A to TFEB-G) Small TFEB	XBP1, PGC-1α, PPARα, MYC; mTORC1 and 14-3-3 binding, lysosomal nutrient sensing, RAG GTPases, RHEB, AMPK, and calcineurin	Regulates autophagy, lysosomal biogenesis, cellular clearance, and lysosomal homeostasis	Biologically central to nutrient sensing, stress adaptation, and lysosome-dependent recovery pathways	[37,59,62,63,65,71,72,73]
**TFE3**	Human Chr X Mouse Chr X	Long isoforms Short isoform	RAG/mTORC1 signaling; ATF4 signaling	Promotes lysosomal biogenesis, autophagy, ER stress adaptation, UPR signaling, and ER-phagy	Highly expressed in placenta, lung, and adrenal gland; involved to stress adaptation and may promote cell death under persistent stress	[27,37,60,97,102]
**TFEC**	Human Chr 7 Mouse Chr 6	TFEC-A, TFEC-B, TFEC-C	IL-4/STAT6 in macrophages	IL-4-regulates TFEC expression in macrophages; positive feedback loop of IL-4-TFEC-IL-4Rα	M2 polarization and allergic asthma-associated immune responses	[37,60,103,105,106]

## 3. Role in Human Pulmonary Diseases

MiT/TFE transcription factors (TFEB, TFE3, MITF, and TFEC) have mainly been studied in neurodegeneration, cancer, and immune regulation, where MiT/TFE proteins control lysosome–autophagy programs, cellular metabolism, and innate immune gene responses. In contrast, their roles in lung biology and pulmonary diseases remain less defined. Most lung-focused work to date has centered on lung cancer and asthma/allergic airway inflammation, where TFEB-linked lysosomal programs and TFEB regulation in airway/immune compartments have been implicated in disease mechanisms. As summarized in Table 2, these emerging observations provide an initial framework for understanding MiT/TFE family members across pulmonary diseases and underscore the need for a broader and more systematic evaluation of their biological and pathological relevance in the lung.

### 3.1. Lung Cancer and Other Pulmonary Tumors

The role of MITF in lung tumors and cancer progression varies depending on the types of cancer cells studied. In lung adenocarcinoma-derived A549 cells, an increase in MITF activity has been associated with resistance to cisplatin (DDP) chemotherapy, as well as enhanced lysosomal biogenesis and autophagy. This indicates a stress-tolerance mechanism that may mitigate the effects of cytotoxic treatments [109]. Hsiao et al. demonstrated higher expression of MITF in low-invasiveness CL1-0 lung adenocarcinoma cells. Both the xenograft mouse model and in vitro studies showed that *MITF* knockdown enhances metastasis and tumorigenesis [110]. Whole-transcriptome analyses and ChIP assays in lung adenocarcinoma suggest that MITF directly binds to the promoter of FZD7, PTGR1, and ANXA1 and acts as a transcriptional repressor to attenuate cell cycle progression, invasion and WNT signaling [110].

Elevated levels of TFEB are linked to poor prognosis in non-small cell lung cancer (NSCLC) through its role in the autophagy–lysosome pathway (ALP), which enhances tumor cell survival and migration and confers resistance to therapeutic interventions [111,112]. In vitro studies using murine lung cancer cells (393P) showed that knockdown of TFEB significantly reversed overexpression of CLEAR genes and Cathepsin D activity caused by TMEM106B overexpression. TMEM106B is a lysosomal transmembrane protein that is a critical driver of lung cancer metastasis [113]. A recent study examining lung adenocarcinoma cohorts identified TFEB as a key factor influencing therapy sensitivity, rather than serving as a consistent pro-resistance lysosomal driver. Elevated levels of TFEB were correlated with improved patient survival rates and a transporter profile (high ABCA1 and low ABCC1 expression) that increases sensitivity to chemotherapy drug cisplatin [114]. In contrast, loss of TFEB led to increased ABCC1-dependent drug efflux and maintenance of mitochondrial ATP/OXPHOS under stress and therefore promotes resistance to platinum-based therapies. Additionally, TFEB was found to support the SREBP2-cholesterol/isoprenoid (IPP) pathway, which enhanced the activation and immune-killing capabilities of Vγ9Vδ2 T-cells through ABCA1-associated metabolite efflux [114]. Thus, in NSCLC, TFEB level seems to affect the sensitivity of cancer cells to treatment. When TFEB levels are low, there is a decreased response to chemotherapy and immune system attacks. A specific transcriptional signature, marked by low TFEB, low ABCA1, and high ABCC1, has been suggested as an indicator of poor outcomes to both chemotherapy and immunotherapy. To counteract this resistance in tumors with low TFEB, it might be possible to restore their vulnerability by targeting the pathways regulated by TFEB.

TFE3 can directly promote pro-proliferative programs by binding the hTERT promoter to support telomerase expression and cell cycle progression [115]. Simultaneously, TFE3 gene fusions characterize rare yet significant subsets of pulmonary tumors where TFE3 is under abnormal regulatory control or forms chimeric transcriptional proteins. YAP1-TFE3 fusion is notably recurrent in clear cell stromal tumors of the lung (CCST-L) [116], perivascular epithelioid cell tumor (PEComas) [117] and pulmonary epithelioid hemangioendothelioma [118], as evidenced by numerous studies and case reports, establishing it as a molecularly distinct entity. Additionally, TFE3 rearrangements appear in exceedingly rare conditions such as pulmonary alveolar soft part sarcoma [119], where molecular confirmation of the ASPSCR1-TFE3 fusion and TFE3 by immunohistochemistry and fluorescence in situ hybridization (IHC/FISH) proves diagnostically valuable [120]. Within epithelioid hemangioendothelioma, cases positive for YAP1-TFE3 fusions have been identified as a distinct molecular subset. Moreover, the spectrum of these fusions is expanding with the discovery of non-canonical TFE3 fusions, such as RREB1-TFE3 [121]. Therapeutic treatment of D-mannose enhanced TFE3-driven lysosomal biogenesis, accelerating the degradation of both wild-type EGF receptor (EGFR) and mutant EGFR (E746-A750 deletion and L858R and T790M mutations) in lysosomes and suppressing NSCLC progression in vitro and in xenograft tumor mouse models [122].

### 3.2. Asthma

In asthma or allergic airway inflammation, the lysosomal–autophagic machinery could act either as a protective mechanism or a harmful one, depending upon the specific cell type, the trigger involved, and the stage of the disease. TFEB is emerging as an important regulator of stress responses in asthma. Using a severe asthma mouse model via intranasal administration of house dust mite (HDM)/c-di-GMP, elevated TFEB activity, achieved by pre-treatment with dexamethasone or trehalose intraperitoneally, dampens NLRP3-dependent inflammatory responses in monocytes and improves disease features [123]. A recent study showed reduced TFEB and other lysosomal gene expressions in airway epithelial cells of ovalbumin induced and HDM-induced asthma mouse models. It also showed that the expression of inflammatory cytokines (NLRP3, IL-1β, and TSLP) was enhanced. Further, microscopy imaging from tissue sections of OVA-treated mice demonstrated SUMO1 expression in airway epithelial cells, and an in vitro co-immunoprecipitation experiment supported increased TFEB SUMOylation upon ovalbumin treatment in BEAS-2B cells. Thus, TFEB SUMOylation inhibits lysosomal biogenesis in airway epithelial cells and promotes asthma development [124]. Another study using OVA- and papain-induced asthma model demonstrated increased TFEB-mediated autophagy with higher ATG5 and LC3 II expressions [125]. Neuropeptide S/NPS receptor expression was found to be high in an asthma mouse model, whereas in vitro studies showed that NPS/NPSR expression induces TFEB expression activity in airway epithelial cells. Thus, NPS/NPSR signaling has been shown to aggravate asthma via a TFEB-dependent autophagy pathway in bronchial epithelial cells, highlighting stimulus- and context-specific outcomes [125]. TFEB has also been reported to influence adaptive immunity by regulating dendritic-cell antigen presentation, including effects on MHC II and co-stimulatory molecules, with downstream consequences for immune balance [126].

### 3.3. COPD/Emphysema

Limited studies have been performed to examine the roles of MiT/TFE family transcription factors in COPD/emphysema. In longitudinal lung tissue sections from patients with COPD–emphysema, nuclear localization of TFEB decreases with disease severity, whereas perinuclear localization of TFEB increases in samples from patients with severe emphysema compared to those with non-emphysema or mild emphysema [127]. Increased perinuclear TFEB was also observed in smokers compared to non-smokers. Notably, cigarette smoke-induced emphysema-like lung histopathology, along with associated autophagy impairment, inflammation, and apoptosis in mice, can be rescued via gemfibrozil-mediated TFEB induction [128]. Separate studies further suggest that TFEB alteration may mediate cigarette smoke-induced lung emphysematous pathology through multiple mechanisms, including increased TFEB oxidation, altered nuclear localization, changes in TFEB expression, and autophagy impairment [129,130]. Treatment of cultured lung alveolar macrophages with cigarette smoke extract induces an inflammatory response accompanied by reduced TFEB activation and impaired autophagy [131]. Moreover, exposure to cigarette smoke extract substantially inhibits TFEB-mediated phagocytosis and bacterial killing in cultured monocyte/macrophages [132]. Although only limited studies have directly linked MiT/TFE activity to COPD/emphysema, key pathological features of COPD/emphysema, including impaired autophagy, mitochondrial damage, oxidative stress, cell senescence, and chronic inflammation, may be associated with dysregulation of MiT/TFE-lysosomal function, which warrants further investigation.

### 3.4. Interstitial Lung Diseases

#### 3.4.1. Birt–Hogg–Dube (BHD) Syndrome

BHD syndrome is an autosomal-dominant disease characterized by facial fibrofolliculomas, renal tumors, and cystic lung disease, which is caused by a loss-of-function mutation in Folliculin (*FLCN*). Up to 90% of individuals with BHD develop pulmonary cysts, with pneumothorax occurring in ~30% [133,134]. Recent findings suggest that FLCN loss disrupts mesenchymal homeostasis and mechanobiology in the lung, resulting in cystic lesions [135,136]. Under nutrient starvation, the FLCN-FNIP complex functions as a GTPase-activating protein, leading to the inactivation of RAG-C/D. A major advance came from the demonstration of a substrate-selective mTORC1 pathway in which TFEB phosphorylation depends strongly on RAG-C/D–mediated amino-acid signaling, explaining how FLCN loss can preferentially dysregulate TFEB control and drive disease phenotypes [4]. In vivo studies showed that constitutive TFEB activation is a key driver of the kidney cyst/cancer-like phenotype in BHD mouse models and that TFEB depletion rescues the renal disease features, positioning TFEB as a central effector downstream of FLCN–RAG–mTORC1 signaling in BHD [15].

Similarly, upon FLCN inactivation, TFE3 becomes dephosphorylated, shuttles to the nucleus and activates its downstream signaling relevant to tumorigenesis [137]. In vitro studies using human fetal lung fibroblasts (MRC-5) demonstrate FLCN inactivation, with a ~ 100-fold decrease in Wnt2 expression and a 33-fold decrease in Wnt7b expression, indicating abnormalities in the WNT pathway’s developmental signals. Silencing the transcription factor TFE3 in FLCN-deficient cells completely reversed this phenotype. Thus, FLCN via TFE3 might play a role in the development of pulmonary cysts associated with BHD [138]. While pulmonary cyst formation in BHD remains an active area of investigation, current evidence limits its mechanistic understanding.

#### 3.4.2. Lymphangioleiomyomatosis (LAM)

LAM is a rare cystic lung disorder characterized by infiltration of the lung by abnormal, smooth-muscle-like LAM cells, associated with a gradual disruption of the tissue architecture. LAM occurs in two forms, sporadic LAM (S-LAM) [139,140] and tuberous sclerosis complex-associated LAM (TSC-LAM), also known as familial LAM (F-LAM) [141,142]. In S-LAM, LAM cells or niche cells usually harbor somatic mutations in the *TSC2* gene, whereas in F-LAM, the underlying issue involves germline disruptions in *TSC1* or *TSC2* genes [143]. LAM predominantly affects women due to risk factors associated with elevated estrogen levels, such as during pregnancy or with external estrogen exposure [144,145,146]. The critical involvement of the mTOR pathway in LAM is highlighted by findings that Sirolimus (an mTORC1 inhibitor) can help stabilize lung function and enhance clinical outcomes during treatment [147,148,149]. Currently, there is no direct evidence reporting MiT/TFE protein involvement in LAM progression. Paradoxically, despite high mTORC1 in LAM cells, TFEB is often nuclear and active. TFEB also promotes mTORC1 activation via Rag GTPases, creating a feedback loop for mTORC1 activation [150]. Recent single-cell RNAseq [151] and spatial transcriptomics studies [152,153,154] using LAMS patient samples also showed MiT/TFE target genes GPNMB, PMEL, and CTSK as differentially expressed genes in LAM core niche cells. Thus, investigating MiT/TFE proteins’ role in LAM could provide insights into how dysregulation of mTORC1 and TFEB/TFE3 alters lysosome-autophagy pathways and metabolic processes in LAM cells and associated niche cells.

#### 3.4.3. Pulmonary Lysosomal Storage Diseases (LSDs)

Pulmonary lysosomal storage diseases (LSDs) are a group of inherited disorders characterized by the accumulation of lysosomal substrates in lung cells due to lysosomal dysfunction [155,156]. This accumulation can lead to patterns such as interstitial lung disease (ILD) and the “storage” phenotypes of alveolar macrophages, with Gaucher disease and Niemann–Pick diseases serving as classic examples [157]. In these disorders, lung involvement may manifest as ILD features observable through imaging, necessitating clinical awareness as part of the disease spectrum [157,158,159]. Mechanistically, TFEB/TFE3 play a crucial role, as TFEB acts as the primary transcriptional regulator of a coordinated lysosomal gene network, known as CLEAR, and becomes activated in response to lysosomal stress or storage conditions. In LSD models, enhancing TFEB activity by treating with sulforaphane, a small-molecule TFEB agonist, promotes lysosomal exocytosis and facilitates cellular clearance, thereby ameliorating storage phenotypes both in vitro and in vivo, which supports the role of TFEB as a functional “lysosomal capacity” switch [160]. Additionally, TFEB and TFE3 influence innate immune cells by promoting lysosomal biogenesis and autophagy, thereby shaping inflammatory responses—an important consideration for pulmonary LSDs, where alveolar macrophages are key drivers of storage and inflammation. Collectively, these studies suggest that profiling and functionally testing TFEB/TFE3 pathways in lung macrophages and epithelial cells from pulmonary LSD patients could elucidate disease variability and aid in developing therapeutic strategies.

**Table 2:** Current evidence for MiT/TFE family members in pulmonary diseases.

Disease	MiT/TFE Family	Target Cell Type	Model System	Effect on MiT/TFE Protein Expression or Activity	Implications	References
Lung cancer	MITF	A549	In vitro and xenograft studies	Higher MITF activity was linked to cisplatin resistance.	Enhanced lysosomal biogenesis, autophagy, and chemotherapy resistance.	[109]
		CL1-0		MITF activity was linked to tumor-suppressive roles.	FZD7/PTGR1/ANXA1 promoter, attenuated cell-cycle progression, invasion, and WNT signaling.	[110]
	TFEB	NSCLCs, 393P	In vitro and human cohort studies	Elevated TFEB was associated with poor prognosis and therapeutic resistance.	CLEAR gene induction and resistance.	[111,112]
				TFEB knockdown reversed CLEAR genes and cathepsin-D overexpression due to TMEM106B^high^ in lung cancer metastasis.	Increased Cathepsin D activity and metastasis.	[113]
				Elevated levels of TFEB were correlated with improved patient survival rates and sensitivity to cisplatin chemotherapy.	Elevated TFEB correlated with ABCA1^high^ and ABCC1^low^ expression.	[114]
Rare pulmonary tumors	TFE3	Pulmonary tumor subsets	Case reports	TFE3 promotes proliferation via hTERT.	hTERT-linked hyperproliferation.	[115]
				YAP1-TFE3 fusions define rare lung tumor subsets such as CCST-L, PEComas, EHE, and PEH.	Fusion protein is constitutively active transcription factor and leads to increased migration and invasiveness.	[116,117,118]
				ASPSCR1-TFE3 fusion in pulmonary alveolar soft-part sarcoma.	Fusion protein directly interacts with the VCP/p97 segregase and super-enhancers (SEs), driving a robust angiogenic program.	[119,120]
				RREB1-TFE3 gene fusion in EHE.	Fusion protein may act as an aberrant transcriptional activator.	[121]
		NSCLC	In vitro studies and xenograft model studies	D-Mannose enhanced TFE3 activity and suppressed NSCLC progression.	Increased lysosomal biogenesis degrades EGFR and EGFR mutants.	[122]
Asthma/ allergic airway inflammation	TFEB	Airway epithelial cells, monocytes, dendritic cell immune context	HDM/c-di-GMP severe asthma mice.	Dexamethasone or Trehalose driven increased TFEB activity was protective in severe asthma.	Increased TFEB activity rescues NLRP3 dependent inflammation.	[123]
			OVA and HDM induced asthma mice and in vitro studies	Reduced TFEB expression and TFEB SUMOylation correlated with asthma development.	Reduced TFEB activity correlated with increased inflammatory cytokines (NLRP3, IL-1β and TSLP). TFEB SUMOylation reduced lysosomal biogenesis.	[124]
			OVA and papain mice and in vitro studies	In this case, increased TFEB-dependent autophagy aggravated asthma.	Increased ATG5, LC3-II, NPS/NPSR signaling driven autophagy.	[125]
COPD/ emphysema	TFEB	Human lung tissue, alveolar macrophages, and monocytes	Case reports, cigarette-smoke emphysema mice and invitro studies	Nuclear TFEB decreased with disease severity, while perinuclear TFEB increased in severe emphysema and in smokers.	TFEB mislocalization associated with autophagy impairment, inflammation and apoptosis was rescued by gemfibrozil-mediated TFEB induction.	[127,128,131,132]
BHD syndrome- associated pulmonary cysts	TFEB	Epithelial, and mesenchymal cells.	BHD mice and in vitro studies	FLCN loss dysregulates TFEB localization; constitutive TFEB activation drives kidney phenotypes in BHD mice models.	Lung-specific evidence is not reported.	
	TFE3	Human fetal lung fibroblasts (MRC-5)	In vitro studies	FLCN inactivation caused TFE3 dephosphorylation/nuclear shuttling, and silencing TFE3 reversed the abnormal WNT-signaling phenotype in FLCN-deficient lung fibroblasts.	Dephosphorylation and nuclear localization of TFE3 and reduced WNT2/WNT7b results in pulmonary cyst development.	[136,138]
LAM	TFEB/ TFE3/ MITF	LAM cells and LAM-niche cells	Human samples scRNA-seq and spatial transcriptomics studies	Nuclear TFEB despite high mTORC1 and increased GPNMB, PMEL, and CTSK in LAM core niche cells.	MiT/TFE-specific evidence is limited.	[152,153,154]
Pulmonary lysosomal storage diseases	TFEB	Alveolar macrophages and epithelial cells	In vitro and in LSD mice	Increased TFEB activity driven by sulforaphane treatment facilitates cellular clearance.	Sulforaphane, a TFEB agonist, induces CLEAR network activation, lysosomal exocytosis, and autophagy/lysosomal biogenesis.	[160]
Acute lung injury and fibrotic remodeling	TFEB	Alveolar Type II cells	LPS-induced ALI rats and in vitro studies	Inhibition of TFEB activity was correlated with inflammation and mitochondrial damage.	Increased TFEB expression led to increased mitophagy, lysosomal function, autophagy flux, and reduced inflammation.	[161]
		Alveolar macrophages	Silica-induced lung injury mice	Trehalose-driven increased TFEB activity prevented fibrotic progression.	Increased TFEB activity improved lysosomal function and autophagy flux.	[162]
		Epithelial and mesenchymal cells	Elastase/cigarette-smoke PiZ mice and in vitro studies.	Increased TFEB activity via lung-directed TFEB gene transfer reduced collagen deposition.	Mechanism is not clear.	[163,164]

### 3.5. Acute Lung Injury and Fibrotic Remodeling

In acute lung injury (ALI) and fibrotic remodeling, TFEB/TFE3 act as stress-response regulators that adjust autophagy/lysosome function, inflammation, and cellular metabolism. Increasing TFEB activity is protective against injury, as demonstrated in LPS-induced ALI mouse models and in vitro experiments. For example, TFEB overexpression reduced inflammation and mitochondrial damage by boosting mitophagy [161]. In the silica-induced lung injury mouse model, where alveolar macrophages develop lysosomal stress and impaired autophagy flux, trehalose-induced TFEB activity improves lysosomal function and autophagy flux and thus reduces fibrotic progression [162]. Another study using a mouse model of elastase- and cigarette smoke-induced emphysema (PiZ model) showed that increased TFEB activity via lung-directed TFEB gene transfer and treatment with autophagy enhancer drug (FLU and CBZ) significantly reduced lung collagen deposition and leukocyte infiltration in mice [163,164]. Thus, TFEB can be protective by restoring cellular clearance and organelle quality control, but its impact on fibrosis and remodeling can vary depending on the tissue and cellular context and is an active area of research. In mechanically stiff environments, TFE3 displays enhanced nuclear localization and transcriptional activity, potentially through altered phosphorylation dynamics and interactions with mechanosensitive signaling pathways [165]. This mechano-regulation positions TFE3 as a transcriptional responder not only to metabolic and lysosomal cues but also to physical properties of the cellular microenvironment, as well as in fibrotic remodeling.

Altogether, the evidence suggests that MiT/TFE activation exerts context-dependent effects on pulmonary disease rather than functioning as a universally protective or pathogenic pathway. In injury, remodeling, and fibrosis-associated settings, activation of TFEB and TFE3 may promote adaptive responses by enhancing lysosomal function, autophagic clearance, and cellular stress resilience, thereby supporting tissue repair and restoration of homeostasis. In contrast, in lung cancer and related tumor contexts, the same MiT/TFE programs may be exploited to sustain tumor cell survival, metabolic flexibility, and resistance to therapy. These observations underscore that the biological consequences of MiT/TFE signaling are shaped by the cellular context, disease stage, and the surrounding niche. Therefore, future studies should move beyond binary interpretations of MiT/TFE function and instead define how different cell types mediate adaptive versus maladaptive processes.

## 4. Discussion and Conclusions

MiT/TFE transcription factors act as central regulators of cellular homeostasis by coordinating lysosome biogenesis and autophagy. They are mainly considered nutrient-responsive on/off switches, but other regulatory mechanisms that control their stability, subcellular localization, and transcriptional activity have been reported in different cellular contexts. For example, TFEB has been shown to be involved in ferritinophagy-mediated iron metabolism and contributes to ferroptosis in injured hepatocytes [166]. TFEB is also able to interact with acetyl-CoA synthetase 2 to locally produce acetyl-CoA for histone H3 acetylation in the TFEB-binding promoter region [166]. This epigenetic modification further promotes its activity in lysosomal biogenesis, autophagy, cell survival, and brain tumorigenesis. Recent studies show that the cGAS–STING pathway engages a TBK1-independent, noncanonical autophagy program that activates TFEB, promotes lysosome biogenesis, and supports endo-lysosomal homeostasis and pathogen clearance [167,168]. The cGAS–STING pathway is an important modulator of pulmonary diseases, such as inflammation, lung injury, fibrosis, and ILDs [169]. Together, these findings also suggest the importance of MiT/TFE in pulmonary diseases.

Despite growing evidence implicating MiT/TFE transcription factors in pulmonary homeostasis and disease, several conceptual and translational questions remain unanswered. Major limitations include validated, disease-relevant biomarkers to identify MiT/TFE pathway activity in human lung disorders. MiT/TFE signaling is a multidimensional process regulated by transcriptional, subcellular localization, post-translational (not discussed in detail) [36,170], nutrient state and cell type-specific outcome. Most evidence relies on total protein abundance, selected downstream targets, or static assessment of nuclear localization. This review summarizes the findings from pulmonary diseases where MiT/TFE factors continue to emerge as critical drivers in pulmonary disorders, including acute lung injury, asthma, lung cancers, LAMS, and LSDs. Therefore, detailed mechanisms and functional roles of MiT/TFE in pulmonary physiology and diseases remain to be explored. Studies should also prioritize cell-type resolved and time-controlled interrogation of MiT/TFE biology in lung disease models by combining inducible, lineage-traced gain- and loss-of-function models for TFE3 and TFEB across key cell compartments (airway epithelium, alveolar macrophages, endothelium, and fibroblast lineages). Future studies will also require integrated framework approaches that combine transcriptional signatures of lysosomal biogenesis and autophagy, post-translational modification-dependent regulatory states, chromatin accessibility, and spatially resolved cell-specific expression patterns to define MiT/TFE activity associated with pulmonary diseases. This will distinguish adaptive MiT/TFE activation that supports cellular homeostasis and repair from maladaptive activation that may reinforce persistent remodeling, tumor survival, or therapy resistance.

## Data Availability

No new data were created or analyzed in this study.

## Abbreviations

AMPK	AMP-activated Protein Kinase.
ATF4	Activating Transcription Factor 4.
BCL2	B-cell Lymphoma 2.
BHD	Birt–Hogg–Dubé Syndrome.
CBZ	Carbamazepine.
CHOP	C/EBP Homologous Protein.
CLEAR	Coordinated Lysosomal Expression and Regulation.
CREB	cAMP Response Element-Binding Protein.
CSF3R	Colony Stimulating Factor 3 Receptor.
CTS	Cathepsin.
CTSK	Cathepsin K.
DDP	Dichlorodiammineplatinum II, Cisplatin.
E-box	Enhancer Box (Consensus CANNTG Motif).
EGFR	Epidermal Growth Factor Receptor.
ERK	Extracellular Signal-Regulated Kinase.
FNIP	Folliculin-Interacting Protein.
GAP	GTPase-Activating Protein.
GATOR	GTPase-Activating Protein Activity Toward Rags.
G-CSF	Granulocyte–Colony Stimulating Factor.
GLI	Glioma-Associated Oncogene.
GPX4	Glutathione Peroxidase 4.
H&E	Hematoxylin and Eosin.
HDM	House Dust Mite.
hTERT	Human Telomerase Reverse Transcriptase.
IHC/FISH	Immunohistochemistry/Fluorescence in Situ Hybridization.
IL-4Rα	Interleukin 4 Receptor Alpha.
IPP	Isoprenoid.
JNK	c-Jun N-terminal Kinase.
LC3	Microtubule-associated Protein 1A/1B-Light Chain 3
MAPK	Mitogen-Activated Protein Kinase.
MAX	MYC-Associated Factor X.
MCOLN1	Mucolipin TRP Cation Channel 1.
MEK	Mitogen-Activated Protein Kinase Kinase.
MITF	Microphthalmia-Associated Transcription Factor.
mTORC1	Mechanistic Target of Rapamycin Complex 1.
NF-κB	Nuclear Factor Kappa-light-chain-enhancer of Activated B cells.
NLRP3	NOD-like Receptor Protein 3.
NPSR	Neuropeptide S Receptor.
NSCLC	Non-Small Cell Lung Cancer.
OVA	Ovalbumin.
OXPHOS	Oxidative Phosphorylation.
PAX3	Paired Box 3 Transcription Factor.
PEComa	Perivascular Epithelioid Cell Tumor.
PGC-1α	Peroxisome Proliferator-Activated Receptor Gamma Coactivator 1-Alpha.
PI3K	Phosphatidylinositol 3-Kinase.
PKA	Protein Kinase A.
PMEL	Premelanosome Protein.
PPARα	Peroxisome Proliferator-Activated Receptor Alpha.
PTEN	Phosphatase and Tensin Homolog.
PTGR1	Prostaglandin Reductase 1.
RAG	Ras-related GTP-binding Protein.
RAS	Rat Sarcoma (Family of Small GTPases).
RTK	Receptor Tyrosine Kinase.
S6K	Ribosomal Protein S6 Kinase.
SLC38A9	Solute Carrier Family 38 Member 9.
SREBP	Sterol Regulatory Element Binding Protein.
SUMO	Small Ubiquitin-like Modifier.
TFE3	Transcription Factor E3.
TFEB	Transcription Factor EB.
TFEC	Transcription Factor EC.
TGF-β	Transforming Growth Factor Beta.
TSC	Tuberous Sclerosis Complex.
TSC1/TSC2	Tuberous Sclerosis Complex Genes 1 and 2.
UBR	Ubiquitin Regulatory Protein.
UPR	Unfolded Protein Response.
v-ATPase	Vacuolar-type H^+^-ATPase.
VEGF	Vascular Endothelial Growth Factor.
WNT	Wingless/Integrated Signaling Pathway.
XBP1	X-box Binding Protein 1.
YAP1	Yes-Associated Protein 1.
